# Three Days Compared to One Day Per Week of Self-Monitoring of Blood Glucose in Mild Gestational Diabetes: A Randomized Trial †

**DOI:** 10.3390/jcm11133770

**Published:** 2022-06-29

**Authors:** Jesrine Gek Shan Hong, Ahmad Firdzaus Mohd. Noor, Peng Chiong Tan

**Affiliations:** Department of Obstetrics and Gynecology, Faculty of Medicine, Universiti Malaya, Jalan Profesor Diraja Ungku Aziz, Kuala Lumpur 50603, Malaysia; jesrine@um.edu.my (J.G.S.H.); ahmadfirdzaus82@gmail.com (A.F.M.N.)

**Keywords:** gestational diabetes, HbA1c, blood sugar profile, self-monitoring blood glucose, birthweight, weight gain

## Abstract

Background: The International Diabetes Federation estimates that 16.2% of livebirths in 2017 were affected by hyperglycemia in pregnancy, with 85.1% due to gestational diabetes mellitus (GDM). Daily blood glucose monitoring compared with alternate day testing in mild GDM is associated with similar pregnancy outcomes. Data are sparse on the ideal frequency for self-monitoring of blood glucose (SMBG) in mild GDM for glycemic control. A higher HbA1c at late pregnancy is associated with adverse pregnancy outcomes. We sought to evaluate three days compared to one day per week of four-point self-monitoring of blood glucose (SMBG) in gestational diabetes mellitus (GDM) controlled by lifestyle changes for glycemic control. Methods: This randomized trial was conducted from February–December 2018. A total of 106 women with lifestyle-controlled GDM were randomized to three days (SMBG3) or one day (SMBG1) per week of four-point (fasting and two-hours post-meal) SMBG. The primary outcome was the change in the HbA1c level at recruitment and 36-weeks gestation within and across trial arms. The student *t*-test was used for between-arm analyses and a paired *t*-test for within-arm analyses. Results: The HbA1c level through pregnancy increased significantly in both trial arms: mean increase of 0.21% ± 0.26%, *p* < 0.001 (SMBG3), and 0.19% ± 0.24%, *p* < 0.001 (SMBG1), but the 0.02% difference across trial arms was not significant (*p* = 0.79). Maternal weight gain (3.1 ± 2.1 kg vs. 3.3 ± 3.0 kg, *p* = 0.72), cesarean delivery (24/52 (48%) vs. 23/53 (43%), RR 1.06, 95% CI: 0.69–1.62, *p* = 0.77), neonatal birthweight (3.1 ± 0.4 kg vs. 3.0 ± 0.4 kg, *p* = 0.53) and neonatal intensive care unit admission (4/52 (8%) vs. 3/53 (6%), RR 1.36, 95% CI: 0.32–5.78, *p* = 0.68) were not significantly different for SMBG3 vs. SMBG1, respectively. Other maternal and neonatal secondary outcomes were not significantly different. Conclusion: In mild GDM, three days compared to one day per week showed a similar HbA1c levels change at 36-weeks gestation. Maternal and neonatal outcomes were also not significantly different. Less frequent monitoring of SMBG as a standard of care in mild GDM deserves further study and consideration.

## 1. Background

The International Diabetes Federation Diabetes Atlas estimated that in 2017, around 21.3 million live births (16.2%) were affected by some form of hyperglycemia in pregnancy, with 86.4% of these due to gestational diabetes mellitus (GDM) [1]. In mild GDM managed by dietary intervention, self-monitoring of blood glucose (SMBG) and insulin therapy reduce the risks of fetal overgrowth, shoulder dystocia, cesarean delivery, hypertensive disorder [2] and serious perinatal morbidity and improve the woman’s health-related quality of life [3]. In both of these mild GDM studies [2,3] 80–90% of women could be managed with SMBG and lifestyle therapy only, without recourse to anti-glycemic drugs [4].

Current guidelines by the American Diabetes Association (ADA) [5], American College of Obstetricians and Gynecologists (ACOG) [6] and the National Institute of Clinical Excellence (NICE) UK [7] recommend SMBG as first-line monitoring for GDM. ACOG guidance suggests daily glucose monitoring four times a day, once after fasting and again after each meal [6], and NICE UK guidance recommends testing fasting and one-hour post-meal blood glucose levels daily [7]. The ADA, in their 2021 report, did not provide guidance on the intensity of SMBG for mild GDM [5], and the current Malaysian 2017 GDM guideline takes a similar position [8].

According to ACOG in their 2018 practice bulletin, there is insufficient evidence to define the optimal frequency of blood glucose testing in women with GDM [6]. A 2009 historical cohort study that compared daily four-point SMBG with weekly single-point fasting office-based testing found a reduction in macrosomia, large for gestational age (LGA) infants and maternal weight gain in the daily SMBG cohort [9]. In contrast, a 2017 randomized trial reported that among patients with well-controlled GDM, alternate day compared to daily four-point SMBG did not increase birthweight, and no significant differences in the need for medical treatment, induction of labor, gestational age at delivery, mode of delivery, rate of pre-eclampsia or shoulder dystocia were found [10]. Alternate day SMBG, which is 3.5 days per week [10], is broadly similar in monitoring frequency to the more intensive arm of our trial, which is at three days per week. In our center, if women with GDM were controlled on lifestyle changes and if the pregnancy was straightforward, the standard monitoring was a four-point blood sugar profile by SMBG one day per week [11].

HbA1c is directly correlated with average glucose levels at a translation rate of 1% HbA1c as equivalent to 1.6 mmol/L estimated average glucose level change [12]. However, in pregnancy, 1% is reported to correspond to 0.67 mmol/L in average blood glucose [13]. Red blood cell turnover increases during pregnancy, accounting for these differences, but the relationship with average blood glucose over the last 6–10 weeks is maintained. The Hyperglycemia and Adverse Pregnancy Outcome (HAPO) study showed associations between increasing fasting, one-hour and two-hour plasma glucose level on the oral glucose-tolerance test (OGTT) and adverse pregnancy outcomes. with no obvious thresholds at which risks increased [14]; HbA1c reflecting average glucose levels was also predictive of these adverse outcomes [15]. Although HbA1c is not recommended as the primary method of monitoring diabetes in pregnancy, nevertheless, the American Diabetes Association (ADA) [5] guidance states that A1C could be used as a secondary measure of glycemic control in pregnancy, after SMBG.

We hypothesized that three days compared to one day per week (four points per day: fasting pre-breakfast and 2 h after each meal) SMBG in women with mild GDM would lower average glycemia and, hence, minimize HbA1c level at 36-weeks gestation.

## 2. Methods

### 2.1. Trial Design

This was a randomized, controlled, clinical intervention trial with an open-label design, comparing three days versus one day per week SMBG in women with GDM controlled through lifestyle modifications. The trial was approved by the Medical Ethics Committee of the University of Malaya Medical Center (date of approval: 29 November 2017; reference number: 2017104-5626) and registered in the International Standard Randomized Controlled Trials Number registry (date of registration: 23 January 2018; reference number: ISRCTN60203274; https://doi.org/10.1186/ISRCTN60203274) prior to enrolment of trial participants. The study was conducted in accordance with the Declaration of Helsinki in University of Malaya Medical Center, with the first participant recruited on 2 February 2018 and the last participant discharged following delivery on 24 December 2018.

### 2.2. Participants

Inclusion criteria were women with GDM (fasting plasma glucose ≥5.1 mmol/L and/or the two-hour level ≥7.8 mmol/L by 75 g OGTT) [8], a normal four-point blood sugar profile (BSP) in the preceding 2 weeks (fasting/pre-prandial <5.3 mmol/L, post-prandial 1 h of <7.8 mmol/L or post-prandial 2 h of <6.7 mmol/L), on lifestyle modification and medical nutritional therapy, 20–30 weeks, singleton gestation and age 18–45 years. Exclusion criteria were women on anti-glycemic drug treatment, with 75 g OGTT criteria that fulfilled Type 2 diabetes (fasting plasma glucose ≥7.0 mmol/L and/or the two-hour level ≥11.1 mmol/L), pre-pregnant hyperglyemia (type 2 diabetes, impaired glucose tolerance or fasting glycemia) or hemoglobin level <8.0 g/dL.

Hospital notes were scrutinized by AFMN in the antenatal clinic of the University of Malaya Medical Center to identify eligible women. Eligible women were approached and provided with the patient information sheet and had oral queries answered. Written informed consent was obtained from all participants. All participants’ relevant demographic and clinical data were transcribed into the case report form. 

### 2.3. Randomization

Participants were randomized in a one-to-one ratio to three days or one day per week SMBG (four points: fasting pre-breakfast and 2 h post-meal at breakfast, lunch and dinner). The randomization sequence (www.random.org, accessed on 25 January 2018) was generated in random blocks of 4 or 8 by a co-investigator (PCT) who was not involved in the recruitment process. Numbered envelope packs were prepared by PCT. The lowest numbered envelope remaining was assigned to the latest recruit. Participants and investigators were not blinded to the allocated intervention. 

### 2.4. Interventions

Venous blood (3 mL) was drawn into an ethylenediaminetetraacetic acid (EDTA) tube for HbA1c analysis at enrolment and at 36-weeks gestation (or at delivery if it was a preterm birth). The blood samples were sent to our hospital laboratory for same-day analysis.

Participants were taught on the use of the glucometer for SMBG. A personal glucometer, glucose strips, lancet needles and alcohol swabs were provided to every participant. 

Women randomized to three days per week four-point SMBG were instructed to perform monitoring two days during the weekdays and one day during the weekend until they delivered. Women randomized to one day per week four-point SMBG would perform monitoring on a weekday in one week and on a weekend in the subsequent week. The four-point SMBGs were pre-breakfast and two-hours post breakfast, lunch and dinner glucometer readings, and participants were instructed to record the readings on an SMBG diary. Participants were told that the fasting target should be <5.3 mmol/L and for 2-h postprandial, <6.7 mmol/L [16].

### 2.5. GDM Care

Standard care for GDM in our center comprised of at least one consult with a dietician for medical nutrition therapy, verbal advice on exercise during pregnancy and gestational weight gain targets as per the Institute of Medicine guidance [17]. Participants were seen every one to four weeks in the antenatal clinic by investigator AFMN, with follow-up frequency indicated by institutional usual practice. At each visit, maternal weight, blood pressure and fundal height were assessed, and a urine dipstick was done for proteinuria and glycosuria. During the visit, the blood sugar profile from the SMBG diary was reviewed, and anti-glycemic treatment was started based on their SMBG readings according to the ACHOIS trial management protocol [3] (two glucometer readings in the preceding two-week period showing a fasting level ≥5.5 mmol/L or the post-prandial level ≥7 mmol/L (≤35-weeks gestation) or post-prandial ≥8.0 mmol/L (>35-weeks gestation) or a solitary ≥9.0 mmol/L). Metformin was used as the first-line therapy [7] at a dose of 500 mg taken at breakfast and dinner. If metformin was insufficient, insulin treatment was instituted with the regimen and dosage of basal bolus fast-acting and longer-acting insulin as guided by the participant’s blood sugar profile on SMBG. 

A fetal ultrasound examination was performed at 36–37-weeks gestation. The timing and mode of delivery was based on our standard practice. In uncomplicated mild GDM, labor induction was recommended at 40-weeks gestation, but for those on anti-glycemic drug therapy, labor induction was offered from 38-weeks gestation [8].

### 2.6. Outcomes Measures

The primary outcomes were the mean difference in HbA1c at recruitment and at 36-weeks gestation within and across trial arms. 

Maternal secondary outcomes included gestational age at delivery (preterm labor <37 weeks), maternal weight at delivery, maternal weight gain, induction of labor, epidural analgesia, mode of delivery, indication for cesarean delivery, delivery blood loss, third- or fourth-degree tear and placenta weight. Secondary neonatal outcomes included birth weight (low birth weight <2.5 kg; macrosomia ≥3.5 kg [18]), umbilical cord arterial pH and base excess at birth, Apgar scores at the first and fifth minutes, reported shoulder dystocia, neonatal birth injury and special care nursery/neonatal intensive care unit admission and indication.

Initiation of anti-glycemic treatment (metformin and/or insulin) were documented. Participants who developed gestational hypertension or pre-eclampsia during follow-up were recorded. Gestational hypertension was defined as BP ≥ 140 mmHg systolic and/or 90 mmHg diastolic on two occasions prior to labor more than six-hours apart, while preeclampsia was gestational hypertension with proteinuria (urine protein–creatinine ratio > 30mg/mmol). Compliance to SMBG was documented, and compliance was defined as 80% or more of the expected number of blood sugar profiles to be performed for the entire study period. 

### 2.7. Sample Size Calculation

Sample size was calculated by using a paired *t*-test [19]. Assuming a mean HbA1c difference of 0.2% from enrolment to 36-weeks gestation and applying a standard deviation of 0.4% in HbA1c values in late pregnancy [20], applying an alpha of 0.05, power of 90% and a one-to-one recruitment ratio, the calculated sample size was 42 for each arm. Factoring in a 20% drop-out rate, 106 (84/0.8) women, in total, were needed.

### 2.8. Statistical Analyses

Data were entered into the SPSS statistical software package (Version 23, IBM, Armonk, NY, USA, SPSS Statistics). The paired *t*-test was used for the analysis of the change in the HbA1c level from recruitment to 36-weeks gestation within the trial arms. The difference in the HbA1c level change from recruitment to 36 weeks across trial arms was analyzed using the student *t*-test. Normally distributed continuous data were analyzed with the student *t*-test, categorical or nominal data with Chi square test (Fisher exact test if small cells <5) and ordinal or non-normal distribution data with a Mann–Whitney U test. Two-sided *p*-values were reported, and *p* < 0.05 was regarded as significant. The primary analysis was on an intention-to-treat basis. 

## 3. Results

**Figure 1:**
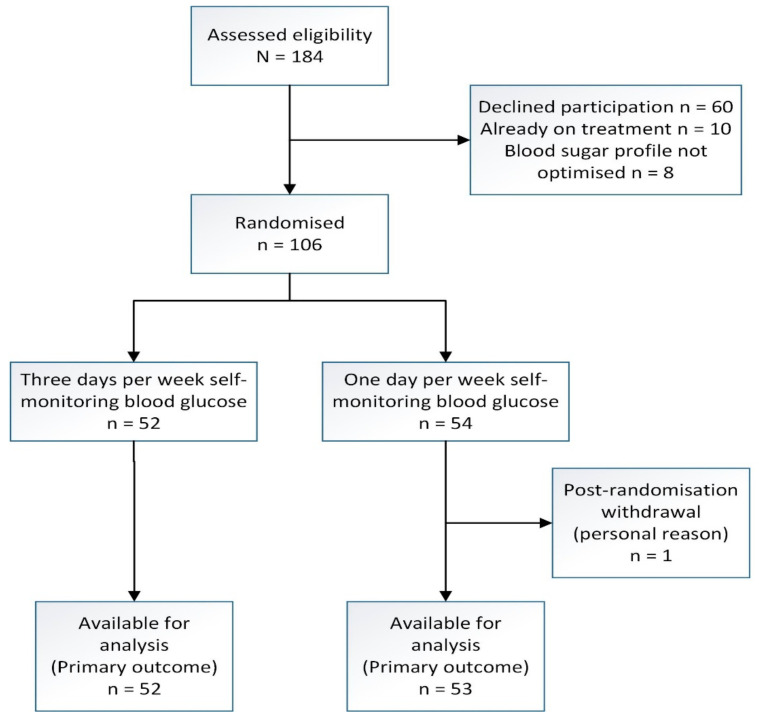
Recruitment flow chart of a randomized trial of three days versus one day per week self-monitoring of blood glucose in mild gestational diabetes mellitus.

Figure 1 depicts the recruitment flow of participants through the study. Of the 184 women diagnosed and managed as GDM in our setting who were identified and approached, 124 women agreed to participate, but 18 women infringed the exclusion criteria, as they were already on anti-glycemic treatment and their four-point BSP was abnormal, which left 106 women to be randomized to SMBG3 (n = 52) and SMBG1 (n = 54). One participant in the SMBG1 arm withdrew from participation after randomization. We included her for analysis based on the intention-to-treat principle. Participants who delivered elsewhere were contacted for their date of delivery, baby’s birth weight and mode of delivery, which were data that we considered would be reliable coming from participants. We stopped recruitment upon exceeding the targeted sample size.

Table 1 lists the characteristics of the trial participants, dichotomized according to their randomization into the SMBG3 or SMBG1 trial arms. Characteristics were not significantly different across the trial arms.

Table 2 shows the primary outcome of change in HbA1c at recruitment to 36-weeks gestation: In both trial arms there were significant increases in HbA1c, with 0.21% ± 0.26% (*p* < 0.001) in the SMBG3 arm and 0.19% ± 0.24% (*p* < 0.001) in the SMBG1 arm (paired *t*-test analysis). However, the mean difference ± standard deviation in HbA1c at recruitment to 36 weeks across the trial arms of 0.21% ± 0.26% versus 0.19% ± 0.24%, *p* = 0.79, was not significantly different (*t*-test analysis).

Table 3 reports the secondary maternal and neonatal outcomes. The number of participants started on treatment for GDM was similar, with 19/52 (36.5%) vs. 18/54 (33.3%), *p* = 0.73, for the SMBG3 vs. SMBG1 arms, respectively. Only 1/106 (1%) of the entire trial population (in the SMBG1 arm) required insulin treatment, while the remainder were solely on metformin. The rate of compliance with self-blood glucose monitoring was 39/48 (81.3%) versus 45/52 (86.5%) (RR 0.94, 95% CI 0.79–1.12, *p* = 0.47) for SMBG3 and SMBG1, respectively. Gestational age at delivery was 38.3 ± 1.3 versus 38.2 ± 1.3 weeks, *p* = 0.91, and maternal gestational weight gain from recruitment to 36-weeks gestation of 3.1 ± 2.1 kg vs. 3.3 ± 3.0 kg, *p* = 0.72, for SMBG3 and SMBG1, respectively, was not significantly different. Preterm delivery (<37 weeks) (6/52 (11.5%) versus 4/53 (7.5%), RR 1.5, 95% CI 0.5–5.1, *p* = 0.49), labor induction (19/52 (36.5%) versus 16/53 (30.2%), RR 1.2, 95% CI 0.7–2.1, *p* = 0.49) and cesarean delivery (24/52 (46.2%) versus 23/53 (43.4%), RR 1.1, 95% CI 0.7–1.6, *p* = 0.77) were also not significantly different for the SMBG3 and SMBG1 arms, respectively. The mean birthweight was 3.1 ± 0.4 kg versus 3.0 ± 0.4 kg, *p* = 0.53; macrosomia (≥3.5 kg) was 8/52 (15.4%) versus 7/53 (13.2%) (RR 1.2, 95% CI 0.5–2.9, *p* = 0.75); low birthweight (<2.5 kg) was 4/52 (7.7%) versus 4/53 (7.5%) (RR 1.0, 95% CI 0.3–3.9, *p* = 0.98) for SMBG3 and SMBG1, respectively. Neonatal admission rates were 4/52 (7.7%) versus 3/53 (5.7%) (RR 1.4, 95% CI 0.3–5.8, *p* = 0.68) for the SMBG3 and SMBG1 arms, respectively.

There were no significant differences reported for other secondary outcomes: Pregnancy-induced hypertension, epidural analgesia, mode of delivery, indication for cesarean delivery, estimated blood loss at delivery, third- or fourth-degree perineal tear, placenta weight, umbilical cord pH at birth, reported shoulder dystocia and neonatal birth injury. No major harms occurred to participants during the trial. 

Post hoc, as the diagnostic threshold for GDM according to our Malaysian national criteria is lower (fasting ≥5.1 and/or 2 h ≥7.8 mmol/L by a 75 g oral glucose tolerance test) [8] compared to the NICE UK (fasting ≥5.6 and/or 2 h ≥7.8 mmol/L) [7] and ADA [5]/IADPSG [21] (fasting ≥5.1 and/or 2 h ≥8.5 mmol/L) criteria, we repeated the outcome analyses restricted to participants who qualified under these respective diagnostic criteria (Supplementary Table S1 for NICE UK [7] and Supplementary Table S2 for ADA [5]/IADPSG [21]). There was no substantive difference for any outcome between the SMBG3 and SMBG1 arms for these post hoc subgroup analyses compared to the main findings of this trial. 

## 4. Discussion

The HbA1c level significantly increased from recruitment (at a mean recruitment gestational age of 28 weeks) to 36 weeks of gestation by 0.21% ± 0.26% (*p* < 0.001) in the SMBG3 and 0.19% ± 0.24% (*p* < 0.001) in the SMBG1 arms. Due to the physiological increases in red blood cell turnover, HbA1c levels fall during normal pregnancy [22], which is in contrast to our data, which showed a significant increase in HbA1c within the trial arms. However, the mean change in HbA1c across trial arms was very similar, demonstrating that despite a three-fold intensity increase in weekly blood glucose monitoring in SMBG3 compared to SMBG1, there was no positive impact on the HbA1c levels over the mean 10-week trial period. A decrease in HbA1c of 0.19% in the CONCEPTT trial [23] was associated with an improved pregnancy outcome, while a higher HbA1c towards the end of pregnancy (36-weeks gestation) is associated with adverse pregnancy outcomes. [3] With appropriate management in GDM, HbA1c declined by a mean of 0.47% per week (range 0.10–1.15%), and the maximum reduction over the four-week study period was 4.3% [24]. In our trial, participants with mild GDM in SMBG3 did not prevent a rise in their HbA1c level nor reduce the increment of HbA1c as compared to those in SMBG1, with the implication that SMBG3 did not improve average glucose in the 6–10 weeks preceding the 36-weeks gestation blood draw for HbA1c. 

A 2021 paper on a pragmatic, randomized clinical trial of gestational diabetes screening, which involved 23,792 women, reported that one-step screening (a 75-g glucose-tolerance test) compared with two-step screening (a 50-g glucose challenge test, followed, if positive, by a 100-g glucose-tolerance test) results in GDM rates of 16.5% and 8.5%, respectively, but no significant between-group differences in the risks of the primary outcomes relating to perinatal and maternal complications [25]. The plausible interpretation is that the additional 8% (presumably with the mildest degree of hyperglycemia) identified and managed as GDM by one-step screening did not appear to improve their pregnancy outcome. Our finding that more intensive monitoring with SMBG in mild GDM did not positively impact on the glycemic status and pregnancy outcome is consistent with the lack of positive impact from the monitoring of the mild or borderline GDM cases in the aforementioned trial.

The ADA, in their 2021 Standards of Medical Care in Diabetes guidance, notes that 70–85% of women diagnosed with GDM under Carpenter–Coustan can control GDM with lifestyle modification alone; it is anticipated that this proportion will be even higher if the lower IADPSG [26] diagnostic thresholds are used [5]. The plausible rationale is that many of the small proportion of women with GDM that required anti-glycemic medication were probably identified soon after diagnosis as their BSP could not be stabilized, and for those controllable by lifestyle change, few were likely to subsequently need anti-glycemic drugs. Hence, SMBG intensity should only have a limited impact on the escalation to drug therapy in mild GDM. Our finding is consistent with this observation [5].

An anti-glycemic agent was used in 36.5% and 33.3% of the SMBG3 and SMBG1 arms, respectively, but only one (1%) participant (in a trial population of 106) in the SMBG1 arm required insulin treatment, and the remainder were on metformin only. The anti-glycemic drug rate is relatively and similarly high across trial arms, as we strictly followed the ACHOIS trial protocol [3] for anti-glycemic intervention, which had an escalation to a first-line insulin rate of 20% in their intervention arm with glucose monitoring instituted. Our first-line anti-glycemic was metformin. Trial data showed that pre-emptive metformin in GDM controlled by lifestyle changes did not positively impact the HbA1c level increment up to 36-weeks gestation compared to the placebo arm, with an anti-glycemic agent use of 17% [11].

The mean birthweight of 3.1 ± 0.4 kg versus 3.0 ± 0.4 kg for SMBG3 compared to SMBG1 was not significantly different; the mean birthweight point estimate was marginally higher amongst the more intensive SMBG3 monitoring. Our findings on birth weight are consistent with a 2017 trial, which reported that among patients with well-controlled GDM, testing blood glucose values every other day did not increase birth weight when compared with women who tested every day [10].

The gestational weight gain was marginally lower, albeit not significant, in the SMBG3 arm (3.1 ± 2.1 kg) compared to 3.3 ± 3.0 kg in the SMBG1 arm. The observed weight gain of just over 3 kg over the mean 10-week trial period indicates reasonable accordance with our guidance of 1–2 kg per month of gestational weight gain in non-obese women. 

The compliance rate with the self-monitoring of blood glucose was higher in the less-intensive SMBG1 arm (86.5% versus 81.3%), although it was not significantly different. This is consistent with a 2017 trial report, which found a higher compliance rate in the every-other-day group as compared with daily group [10].

A 2009 report showed that daily blood glucose self-monitoring compared with weekly office-based testing in women with diet-treated gestational diabetes was associated with a reduction in the incidence of macrosomia [9], and this is in keeping with Langer et al., who reported, in 1994, a reduction in macrosomia with an increased intensity of glycemic monitoring [27]. In our trial, the birthweight and macrosomia rate (≥3.5 kg) were similar in both trial arms. More frequent glucose monitoring in mild GDM did not materially improve the neonatal outcomes, corroborating a 2002 trial report, which compared four-times-daily SMBG to periodic monitoring at prenatal visits in women with diet-controlled GDM and showed no significant difference in pregnancy or neonatal outcomes [28]. A more recent trial in 2017 reported that among patients with well-controlled GDM, testing blood glucose values every other day compared to daily resulted in no significant differences with regards to the need for medical treatment; induction; gestational age at delivery; mode of delivery; rate of pre-eclampsia; shoulder dystocia; birth weight and neonatal outcomes, including neonatal hypoglycemia [10]. A 2017 Cochrane systematic review and meta-analysis on different methods and settings for glucose monitoring for gestational diabetes drew on 11 randomized controlled trials, which included 1272 women, and it suggested no clear differences for the primary outcomes or other secondary outcomes assessed in the review [29]. Our findings and these more recent reports [10,25,29] should point to a more nuanced approach than guidelines [7] that recommend a blanket daily SMBG approach to all GDM cases. The GDM controlled by lifestyle change category, which comprises at least 70% of GDM cases [5], could benefit from de-intensification to one day per week of SMBG monitoring without increasing adverse pregnancy outcomes according to our data. It is plausible that further de-intensification in SMBG for mild GDM may be non-inferior, as a two-step compared to one-step GDM screening trial, which essentially halved the GDM diagnosis rate from 16.5% to 8.5%, showed no difference in the pregnancy outcome, despite no monitoring for the “undetected” cases from the two-step screening [25].

## 5. Strengths and Limitations

With regards to the strengths, our trial population was a high-risk, multi-ethnic Asian population. Therefore, we believe that our findings will be generalizable to mild GDM cases across similar populations and that they should also be generalizable to lower risk populations. Our trial was powered to our primary outcome of detecting a small (0.2%) change in the HbA1c level. The outcome data were replete with a high (more than 80%) compliance rate to SMBG and drop-out rates well below the 20% anticipated in our sample size calculation. All participants were managed antenatally by one investigator to minimize care variation. 

Regarding limitations, although our study was powered to the effect that we anticipated, the actual across-trial arms effect was smaller. Hence, our study became underpowered. However, given the very modest difference we identified, a powered study on an HbA1c change of that magnitude may have little clinical relevance. Our trial was conducted within a single center, and routine outpatient antenatal follow up was by a single investigator, potentially reducing external validity. Our trial is not powered to directly address clinical outcomes. 

## 6. Conclusions

In mild GDM, four-point self-monitoring of blood glucose three days per week compared to one day per week showed very similar HbA1c levels at 36-weeks gestation and no significant difference in other secondary maternal and neonatal outcomes. Three days per week self-monitoring of blood glucose is not superior to one day per week in mild GDM. 

## Figures and Tables

**Table 1:** Characteristics of trial participants with mild gestational diabetes ^a^ randomized to the self-monitoring of blood glucose of three days (SMBG3) or one day per week (SMBG1) arms.

Characteristics	SMBG3 N = 52	SMBG1 N = 54	*p*-Value
Age (years)	31.6 ± 4.2	33.5 ± 4.2	0.06
Parity	0.5 [0–1.0]	2.0 [0–2.0]	0.004
0	26 (50.0%)	16 (29.6%)	0.009
1	14 (26.9%)	10 (18.5%)	
≥2	12 (23.1%)	28 (51.9%)	
Nullipara	26 (50.0%)	16 (29.6%)	0.047
Multipara	26 (50.0%)	38 (70.4%)	
Gestational age at recruitment (weeks)	28.3 ± 3.8	27.6 ± 3.3	0.33
Gestational age at recruitment (weeks) ≤24	7 (13.5%)	9 (16.7%)	0.65
Gestational age at recruitment (weeks) >24	45 (86.5%)	45 (83.3%)	
Gestational age at diagnosis of gestational diabetes (weeks)	20.7 ± 6.1	19.2 ± 5.6	0.21
Oral glucose tolerance test			
Fasting glucose (mmol/L)	4.8 ± 0.6	4.8 ± 0.7	0.84
≤5.0 mmol/L	36 (69.2%)	32 (59.3%)	0.48
5.1–5.5 mmol/L	11 (21.2%)	17 (31.5%)	
≥5.6 mmol/L	5 (9.6%)	5 (9.3%)	
Two-hour glucose (mmol/L)	8.1 ± 1.2	7.8 ± 1.3	0.15
≤7.7 mmol/L	9 (17.3%)	15 (27.8%)	0.43
7.8–8.4 mmol/L	22 (42.3%)	20 (37%)	
≥8.5 mmol/L	21 (40.4%)	18 (33.3%)	
GDM based on NICE UK ^b^	46 (88.5%)	43 (79.6%)	0.22
GDM based on IADPSG ^c^	35 (67.3%)	35 (64.8%)	0.79
HbA1c (%) at recruitment	5.2 [5.0–5.4]	5.2 [4.9–5.5]	0.93
Hemoglobin level (g/dL)	11.7 ± 1.3	11.7 ± 1.3	0.97
Height (meters)	1.58 ± 0.06	1.57 ± 0.06	0.22
	1.58 [1.55–1.63]	1.56 [1.53–1.62]	0.18
Weight (kg) at recruitment	72.1 ± 12.5	71.7 ± 13.7	0.88
Body mass index (kg/m^2^) pre-pregnancy	26.2 ± 5.1	26.9 ± 5.5	0.53
Body mass index (kg/m^2^) at recruitment	28.7 ± 4.7	29.1 ± 5.2	0.73
<25	13 (25.0%)	9 (16.7%)	0.55
25.0–29.9	17 (32.7%)	21 (38.9%)	
≥30	22 (42.3%)	24 (44.4%)	
Previous cesarean delivery	12 (23.1%)	16 (29.6%)	0.44
Previous macrosomic baby (≥4 kg)	0 (0%)	1 (1.9%)	0.32
Ethnicity			
Malay	33 (63.5%)	48 (88.9)	0.09
Chinese	16 (30.8%)	5 (9.3%)	
Others ^d^	3 (5.8%)	1 (1.9%)	

Data are represented as the mean ± standard deviation, median [interquartile range] and number (%). Analyses were by *t*-test for the means of continuous data, Mann–Whitney U test for non-parametric data (assessed by the Kolmogorov–Smirnov test) and the Chi-square test for categorical data. ^a^ Based on Malaysian national guideline criteria: oral glucose tolerance test fasting ≥ 5.1 and/or 2 h ≥ 7.8 mmol/L. ^b^ GDM based on NICE UK criteria: oral glucose tolerance test fasting ≥ 5.6 and/or 2 h ≥ 7.8 mmol/L. ^c^ GDM based on IADPSG criteria: oral glucose tolerance test fasting ≥ 5.1 and/or 2 h ≥ 8.5 mmol/L. ^d^ Others represent 1 Indian, 1 Sabah native and 1 Filipino in SMBG3 and 1 Filipino in SMBG1.

**Table 2:** Primary outcome of trial participants randomized to the self-monitoring of blood glucose of three days (SMBG3) or one day per week (SMBG1) arms.

Outcomes	SMBG3 N = 52	SMBG1 N = 53	RR (95% CI)	*p*-Value
HbA1c (%) at recruitment	5.21 ± 0.36	5.18 ± 0.46		0.68
HbA1c (%) at 36-weeks gestation	5.42 ± 0.38	5.38 ± 0.40		0.58
HbA1c ≥ 6.0% at 36-weeks gestation ^a^	4 (7.7%)	3 (5.7%)	1.36 (0.32–5.78)	0.67
HbA1c ≥ 6.5% at 36-weeks gestation ^b^	1 (1.9%)	0 (0%)		0.31
Mean change in HbA1c: Recruitment to 36 weeks	+0.21 ± 0.26	+0.19 ± 0.24		0.79 ^c^
	*p* < 0.001 ^d^	*p* < 0.001 ^d^		

Data are represented the mean ± standard deviation. Analyses were by a paired or independent student *t*-test for means. ^a^ Target HbA1c was based on the American Diabetic Association recommendations. ^b^ Target HbA1c was based on the NICE UK recommendations. ^c^ Analyzed by an independent *t*-test across trial arms. ^d^ Analyzed by a paired - test within the trial arm.

**Table 3:** Secondary outcomes of trial participants randomized to the self-monitoring of blood glucose of three days (SMBG3) or one day per week (SMBG1) arms.

Outcomes	SMBG3 N = 52	SMBG1 N = 53	RR (95% CI)	*p*-Value
**Maternal outcomes**				
Treatment for Gestational diabetes	19 (36.5%)	18 (33.3%)	1.10 (0.65–1.84)	0.73
Metformin only	19 (100%)	17 (94.4%)	1.06 (0.95–1.18)	0.30
Insulin	0 (0%)	1 (5.6%)		
Gestational hypertension	1 (1.9%)	4 (7.4%)	0.26 (0.03–2.25)	0.18
Pre-eclampsia	0 (0%)	3 (5.6%)		0.17
	n = 48	n = 52		
Compliance to SMBG ^a^	39 (81.3%)	45 (86.5%)	0.94 (0.79–1.12)	0.47
Gestational age at delivery (weeks)	38.3 ± 1.3	38.2 ± 1.3		0.91
Preterm labor (<37 weeks)	6 (11.5%)	4 (7.5%)	1.53 (0.46–5.11)	0.49
Weight at delivery (kg)	75.2 ± 12.7	74.9 ± 13.7		0.90
Maternal weight gain (kg)	3.1 ± 2.1	3.3 ± 3.0		0.72
Body mass index at delivery (kg/m^2^)	29.9 ± 4.7	30.4± 5.1		0.64
Induction of labor	19 (36.5%)	16 (30.2%)	1.21 (0.70–2.09)	0.49
Prostaglandin	6 (31.6%)	10 (62.5%)	0.51(0.24–1.08)	0.07
Foley catheter	13 (68.4%)	6 (37.5%)	1.83 (0.90–3.68)	
Oxytocin use in labor	30 (57.7%)	25 (47.2%)	1.22 (0.85–1.77)	0.28
Epidural analgesia in labor	10 (19.2%)	4 (7.5%)	2.55 (0.85–7.61)	0.08
Mode of delivery				0.52
Spontaneous vaginal delivery	25 (48.1%)	29 (54.7%)	0.88 (0.61–1.28) ^a^	0.49 ^b^
Operative vaginal delivery	3 (5.8%)	1 (1.9%)		
Cesarean delivery	24 (46.2%)	23 (43.4%)	1.06 (0.69–1.62) ^b^	0.77 ^c^
Emergency	16 (66.7%)	15 (65.2%)	1.02 (0.68–1.54)	0.92
Elective	8 (33.3%)	8 (34.8%)	0.96 (0.43–2.12)	
Indications for emergency cesarean	n = 16	n = 15		
Failure to progress ^d^	9 (56.3%)	6 (40.0%)		0.46
Non-reassuring fetal status	5 (31.3%)	5 (33.3%)		
Previous cesarean deliver y(In labor)	2 (12.5%)	2 (13.3%)		
Malpresentation	0 (0%)	2 (13.3%)		
Indications for elective cesarean	n = 8	n = 8		
Previous cesarean delivery	5 (62.5%)	7 (87.5%)		0.32
Breech presentation	1 (12.5%)	1 (12.5%)		
Placenta previa	2 (25.0%)	0 (0%)		
Estimated blood loss at delivery (mL)	300 [300–400]	300 [250–400]		0.80
Postpartum hemorrhage (≥500 mL)	12 (23.1%)	11 (20.8%)	1.11(0.54–2.29)	0.77
Postpartum hemorrhage (≥1000 mL)	0 (0%)	3 (5.7%)		0.08
Third-or fourth degree perineal tear				
	0 (0%)	0 (0%)		
Placenta weight (g)	n = 33	n = 36		
	581.4 ± 90.9	558.5 ± 87.2		0.29
**Neonatal outcomes**				
Birthweight (kg)	3.1 ± 0.4	3.0 ± 0.4		0.53
Birthweight ≥ 4.0 kg	0 (0%)	1 (1.9%)		0.32
Birthweight ≥ 3.5 kg	8 (15.4%)	7 (13.2%)	1.17 (0.46–2.98)	0.75
Birthweight < 2.5 kg	4 (7.7%)	4 (7.5%)	1.02 (0.27–3.86)	0.98
Neonatal birth injury	0 (0%)	0 (0%)		
Shoulder dystocia	0 (0%)	0 (0%)		
Apgar score at 1 min	9 [9–9]	9 [9–9]		0.69
Apgar score at 5 min	10 [10–10]	10 [10–10]		0.39
Neonatal admission	4 (7.7%)	3 (5.7%)	1.36 (0.32–5.78)	0.68
Indication of neonatal admission				
Presumed sepsis	1 (25%)	3 (100%)		0.27
Prematurity	1 (25%)	0 (0%)		
Fetal anemia	1 (25%)	0 (0%)		
Meconium aspiration syndrome	1 (25%)	0 (0%)		
Cord arterial blood pH	n = 47	n = 47		
	7.31 ± 0.06	7.30 ± 0.09		0.58
Cord arterial blood base excess	n = 43 −2.93 ± 2.53	n = 45 −3.45 ± 2.84		0.36

Data expressed as mean ± standard deviation, median [interquartile range] or number (%). Analyses by Student *t*-test for continuous data, Fisher’s exact test for 2 × 2 categorical datasets, Chi Square test for larger than 2 × 2 categorical datasets and Mann–Whitney U test for non-parametric data (assessed by Kolmogorov-Smirnov test) or ordinal data. The two-sided *p* was <0.05 for all variables. ^a^ Compliance to self-monitoring of blood glucose (SMBG) was defined as ≥80% of the expected amount of self-monitoring of blood glucose to be performed for the entire study period. ^b^ Spontaneous vaginal delivery compared to operative delivery (instrumental vaginal and cesarean delivery). ^c^ Cesarean delivery compared to vaginal delivery (spontaneous vaginal and instrumental vaginal delivery). ^d^ Failure to progress included a poor progress of labor, failed induction of labor and secondary arrest.

## Data Availability

The datasets used and/or analyzed during the current study are available from the corresponding author on reasonable request.

## Supplementary Materials

The following supporting information can be downloaded at: https://www.mdpi.com/article/10.3390/jcm11133770/s1, Table S1: Post hoc analyses. GDM based on NICE UK criteria. Table S2: Post hoc analyses. GDM based on IADPSG criteria.

## Abbreviations

GDM	Gestational diabetes mellitus
HbA1c	Glycated hemoglobin
SMBG1	One day per week self-monitoring of blood glucose
SMBG3	Three days per week self-monitoring of blood glucose
