# Supplementary material for: Three Days Compared to One Day Per Week of Self-Monitoring of Blood Glucose in Mild Gestational Diabetes: A Randomized Trial
Source: J Clin Med. 2022 Jun 29;11(13):3770. doi: 10.3390/jcm11133770 (PMC9267312; doi:10.3390/jcm11133770)
Supplement: Supplementary file 1 [file jcm-11-03770-s001.zip › jcm-1753374-supplementary.pdf]

Supplementary Table S1: Post hoc analyses. GDM based on NICE UK criteria[a]

A. Primary outcome of trial participants randomized to self-monitoring of blood glucose of three days (SMBG3) or one day per week (SMBG1) arm.

Outcomes	SMBG3<br>N = 46	SMBG1<br>N = 43	RR (95% CI)	P value
HbA1c (%) at recruitment	5.22 ± 0.37	5.14 ± 0.49		0.41
	n = 46	n = 42		
HbA1c (%) at 36 weeks gestation	5.42 ± 0.38	5.32 ± 0.37		0.22
HbA1c ≥6.0% at 36 weeks gestation[b]	4 (8.7%)	1 (2.4%)	3.65 (0.43-31.39)	0.20
HbA1c ≥6.5% at 36 weeks gestation[c]	1 (2.2%)	0 (0%)		0.34
Mean change in HbA1c: Recruitment to 36 weeks	+0.21 ± 0.26	+0.18 ± 0.25		0.58[d]
	P < 0.001[c]	P < 0.001[e]		

Data are represented mean ± standard deviation. Analyses were by paired or independent student *t* test for means

[a] GDM based on NICE UK criteria: oral glucose tolerance test fasting ≥5.6 and/or 2-hour ≥7.8 mmol/L

[b] Target HbA1c based on American Diabetic Association recommendations

[c] Target HbA1c based on NICE UK recommendations

[d] Analyzed by independent t test across trial arms.

[e] Analyzed by paired t test within trial arm.

B. Secondary outcomes of trial participants randomized to self-monitoring of blood glucose of three days (SMBG3) or one day per week (SMBG1) arm.

Outcomes	SMBG3<br>N = 46	SMBG1<br>N = 43	RR (95% CI)	P value
**Maternal outcomes**				
Treatment for gestational diabetes	19 (41.3%)	14 (32.6%)	1.27 (0.73-2.20)	0.39
Metformin only	19 (100%)	13 (92.9%)	1.08 (0.93-1.25)	0.24
Insulin	0 (0%)	1 (7.1%)		
Gestational hypertension	0 (0%)	4 (9.3%)		0.03
Pre-eclampsia	0 (0%)	3 (7.0%)		.[a]
	**n = 42**	**n = 41**		
Compliance to SMBG[b]	35 (83.3%)	37 (90.2%)	0.92 (0.78-1.09)	0.35
	**n = 46**	**n = 42**		
Gestational age at delivery (weeks)	38.2 ± 1.4	38.3 ± 1.3		0.89
Preterm labor (<37 weeks)	5 (10.9%)	3 (7.1%)	1.58 (0.36-7.08)	0.54
Weight at delivery (kg)	74.7 ± 13.3	72.0 ± 13.1		0.35
Maternal weight gain (kg)	3.3 ± 2.1	3.4 ± 3.1		0.77
Body mass index at delivery (kg/m^2)	29.7 ± 4.9	29.7± 5.2		0.99

Induction of labor	18 (39.1%)	14 (33.3%)	1.17 (0.67-2.05)	0.57
Prostaglandin	6 (33.3%)	8 (57.1%)	0.58(0.26-1.29)	0.18
Foley catheter	12 (66.7%)	6 (42.9%)	1.56 (0.78-3.09)	
Oxytocin use in labor	27 (58.7%)	22 (52.4%)	1.12 (0.77-1.63)	0.55
Epidural analgesia in labor	10 (21.7%)	4 (9.5%)	2.28 (0.77-6.73)	0.12
Mode of delivery				0.64
Spontaneous vaginal delivery	24 (52.2%)	22 (52.4%)	0.99 (0.67-1.48)[b]	0.99[c]
Operative vaginal delivery	3 (6.5%)	1 (2.4%)		
Cesarean delivery	19 (41.3%)	19 (45.2%)	0.91 (0.57-1.47)[c]	0.71[d]
Emergency	13 (68.4%)	12 (63.2%)	1.08 (0.68-1.71)	0.73
Elective	6 (31.6%)	7 (36.8%)	0.86 (0.35-2.08)	
Indications for emergency cesarean	**n = 13**	**n = 12**		
Failure to progress[e]	8 (61.5%)	5 (41.7%)		0.62
Non-reassuring fetal status	3 (23.1%)	4 (33.3%)		
Previous caesarean delivery (In labor)	2 (15.4%)	2 (16.7%)		
Malpresentation	0 (0%)	1 (8.3%)		
Indications for elective cesarean	**n = 6**	**n = 7**		
Previous caesarean delivery	4 (66.7%)	6 (85.7%)		0.42
Breech presentation	1 (16.7%)	1 (14.3%)		
Placenta previa	1 (16.7%)	0 (0%)		
Estimated blood loss at delivery (ml)	300 [300-400]	300 [250-400]		0.59
Postpartum hemorrhage (≥ 500mL)	11 (23.9%)	10 (23.8%)	1.00(0.48-2.12)	0.99
Postpartum hemorrhage (≥1000mL)	0 (0%)	3 (7.1%)		0.08
Third-or fourth degree perineal tear	**n = 27**	**n = 23**		
	0 (0%)	0 (0%)		
Placenta weight (gram)	**n = 32**	**n = 28**		
	580.8 ± 92.3	544.1 ± 85.6		0.12
**Neonatal outcomes**	**n = 46**	**n = 42**		
Birthweight (kg)	3.1 ± 0.4	3.0 ± 0.5		0.38
Birthweight ≥4.0kg	0 (0%)	1 (2.4%)		0.29
Birthweight ≥3.5kg	5 (10.9%)	5 (11.9%)	0.91 (0.28-2.93)	0.88
Birthweight <2.5kg	3 (6.5%)	4 (9.5%)	0.69 (0.16-2.88)	0.60
Neonatal birth injury	0 (0%)	0 (0%)		
Shoulder dystocia	0 (0%)	0 (0%)		
Apgar score at 1 minute	9 [9-9]	9 [9-9]		0.82
Apgar score at 5 minutes	10 [10-10]	10 [10-10]		0.47
Neonatal admission	4 (8.7%)	3 (7.1%)	1.36 (0.32-5.78)	0.79
Indication of neonatal admission				
Presumed sepsis	1 (25%)	3 (100%)		0.27

Prematurity	1 (25%)	0 (0%)	
Fetal anemia	1 (25%)	0 (0%)	
Meconium aspiration syndrome	1 (25%)	0 (0%)	
Cord arterial blood pH	**n = 41**	**n = 37**	
	7.31 ± 0.06	7.30 ± 0.09	0.33
Cord arterial blood base excess	**n = 37**	**n = 36**	
	-3.03 ± 2.61	-3.64 ± 2.91	0.35

Data expressed as mean ± standard deviation, median [interquartile range] or number (%). Analyses by Student t test for continuous data, Fisher's exact test for 2x2 categorical datasets, Chi Square test for larger than 2x2 categorical datasets and Mann Whitney U test for non-parametric data (assessed by Kolmogorov-Smirnov test) or ordinal data. 2-sided P<0.05 for all variables.

[a] No statistics are computed because SMBG1 is a constant

[b] Compliance to self-monitoring of blood glucose (SMBG) is defined as ≥ 80% of expected number of self-monitoring of blood glucose to be performed for the entire study period

[c] Spontaneous vaginal delivery compared to operative delivery (instrumental vaginal and caesarean delivery)

[d] Cesarean delivery compared to vaginal delivery (spontaneous vaginal and instrumental vaginal delivery)

[e] Failure to progress includes poor progress of labor, failed induction of labor and secondary arrest

Supplementary Table S2: Post hoc analyses. GDM based on IADPSG criteria[a]

A. Primary outcome of trial participants randomized to self-monitoring of blood glucose of three days (SMBG3) or one day per week (SMBG1) arm.

Outcomes	SMBG3<br>N = 35	SMBG1<br>N = 35	RR (95% CI)	P value
HbA1c (%) at recruitment	5.22 ± 0.41	5.31 ± 0.42		0.36
HbA1c (%) at 36 weeks gestation	5.45 ± 0.43	5.49 ± 0.36		0.68
HbA1c ≥6.0% at 36 weeks gestation[b]	4 (11.4%)	3 (8.6%)	1.33 (0.32-5.53)	0.69
HbA1c ≥6.5% at 36 weeks gestation[c]	1 (2.9%)	0 (0%)		0.31
Mean change in HbA1c: Recruitment to 36 weeks	+0.22 ± 0.29	+0.17 ± 0.25		0.43[d]
	P < 0.001[e]	P < 0.001[e]		

Data are represented mean ± standard deviation. Analyses were by paired or independent student *t* test for means

[a] GDM based on IADPSG criteria: oral glucose tolerance test fasting ≥5.1 and/or 2-hour ≥8.5 mmol/L

[b] Target HbA1c based on American Diabetic Association recommendations

[c] Target HbA1c based on NICE UK recommendations

[d] Analyzed by independent t test across trial arms.

[e] Analyzed by paired t test within trial arm.

B. Secondary outcomes of trial participants randomized to self-monitoring of blood glucose of three days (SMBG3) or one day per week (SMBG1) arm.

Outcomes	SMBG3<br>N = 35	SMBG1<br>N = 35	RR (95% CI)	P value
**Maternal outcomes**				
Treatment for gestational diabetes	11 (31.4%)	15 (42.9%)	0.73 (0.39-1.37)	0.32
Metformin only	11 (100%)	14 (93.3%)	1.07 (0.94-1.23)	0.38
Insulin	0 (0%)	1 (6.7%)		
Gestational hypertension	1 (2.9%)	3 (8.6%)	0.33 (0.04-3.05)	0.30
Pre-eclampsia	0 (0%)	2 (5.7%)		0.25
	**n = 33**	**n = 34**		
Compliance to SMBG[a]	26 (78.8%)	29 (85.3%)	0.92 (0.74-1.16)	0.49
Gestational age at delivery (weeks)	38.3 ± 1.5	38.3 ± 1.2		0.83
Preterm labor (<37 weeks)	4 (11.4%)	2 (5.7%)	2.00 (0.39-10.22)	0.39
Weight at delivery (kg)	75.9 ± 13.1	76.9 ± 14.0		0.77
Maternal weight gain (kg)	2.9 ± 1.9	3.1 ± 2.8		0.83
Body mass index at delivery (kg/m^2)	30.3 ± 4.6	31.0 ± 5.3		0.53
Induction of labor	13 (37.1%)	12 (34.3%)	1.08 (0.58-2.03)	0.80
Prostaglandin	4 (30.8%)	10 (83.3%)	0.37 (0.16-0.88)	0.08

Foley catheter	9 (69.2%)	2 (16.7%)	4.15 (1.11-15.49)	
Oxytocin use in labor	22 (62.9%)	17 (48.6%)	1.29 (0.85-1.98)	0.23
Epidural analgesia in labor	5 (14.3%)	3 (8.6%)	1.67 (0.43-6.45)	0.45
Mode of delivery				0.28
Spontaneous vaginal delivery	17 (48.6%)	21 (60%)	0.81 (0.52-1.25)[b]	0.34[b]
Operative vaginal delivery	2 (5.7%)	0 (0%)		
Cesarean delivery	16 (45.7%)	14 (40.0%)	1.14 (0.66-1.97)[c]	0.63[c]
Emergency	12 (75%)	9 (64.3%)	1.17 (0.72-1.89)	0.52
Elective	4 (25%)	5 (35.7%)	0.70 (0.23-2.11)	
Indications for emergency cesarean	**n = 12**	**n = 9**		
Failure to progress[d]	5 (41.7%)	3 (33.3%)		0.67
Non-reassuring fetal status	5 (41.7%)	4 (44.4%)		
Previous caesarean delivery (In labor)	2 (16.7%)	1 (11.1%)		
Malpresentation	0 (0%)	1 (11.1%)		
Indications for elective cesarean	**n = 4**	**n = 5**		
Previous cesarean delivery	2 (50%)	5 (100%)		0.49
Breech presentation	1 (25%)	0 (0%)		
Placenta previa	1 (25%)	0 (0%)		
Estimated blood loss at delivery (ml)	300 [300-400]	300 [250-400]		0.61
Postpartum hemorrhage (≥500mL)	9 (25.7%)	9 (25.7%)	1.00(0.45-2.22)	1.00
Postpartum hemorrhage (≥1000mL)	0 (0%)	2 (5.7%)		0.15
Third-or fourth degree perineal tear	**n = 19**	**n = 21**		
	0 (0%)	0 (0%)		
Placenta weight (gram)	**n = 22**	**n = 26**		
	575.7 ± 93.4	555.6 ± 82.6		0.43
**Neonatal outcomes**	**n = 35**	**n = 35**		
Birthweight (kg)	3.1 ± 0.4	3.1 ± 0.4		0.82
Birthweight ≥4.0kg	0 (0%)	1 (2.9%)		0.31
Birthweight ≥3.5kg	6 (17.1%)	5 (14.3%)	1.20 (0.40-3.57)	0.74
Birthweight <2.5kg	3 (8.6%)	2 (5.7%)	1.50 (0.27-8.43)	0.64
Neonatal birth injury	0 (0%)	0 (0%)		
Shoulder dystocia	0 (0%)	0 (0%)		
Apgar score at 1 minute	9 [9-9]	9 [9-9]		0.18
Apgar score at 5 minutes	10 [10-10]	10 [10-10]		0.31
Neonatal admission	3 (8.6%)	2 (5.7%)	1.50 (0.27-8.43)	0.64
Indication of neonatal admission				
Presumed sepsis	0 (0%)	2 (100%)		0.17
Prematurity	1 (33.3%)	0 (0%)		
Fetal anemia	1 (33.3%)	0 (0%)		
Meconium aspiration syndrome	1 (33.3%)	0 (0%)		

Cord arterial blood pH	**n = 34**	**n = 31**	
	7.31 ± 0.06	7.32 ± 0.06	0.61
Cord arterial blood base excess	**n = 30**	**n = 30**	
	-3.11 ± 2.83	-3.13 ± 2.72	0.98

Data expressed as mean ± standard deviation, median [interquartile range] or number (%). Analyses by Student t test for continuous data, Fisher's exact test for 2x2 categorical datasets, Chi Square test for larger than 2x2 categorical datasets and Mann Whitney U test for non-parametric data (assessed by Kolmogorov-Smirnov test) or ordinal data. 2-sided P<0.05 for all variables.

[a] Compliance self-monitoring of blood glucose (SMBG) is defined as ≥ 80% of expected number of self-monitoring of blood glucose to be performed for the entire study period

[b] Spontaneous vaginal delivery compared to operative delivery (instrumental vaginal and cesarean delivery)

[c] Cesarean delivery compared to vaginal delivery (spontaneous vaginal and instrumental vaginal delivery)

[d] Failure to progress includes poor progress of labor, failed induction of labor and secondary arrest
